# Sensory dynamics transformation into effective motor behavior

**DOI:** 10.1186/1471-2202-14-S1-F2

**Published:** 2013-07-08

**Authors:** Roberto Latorre, Rafael Levi, Pablo Varona

**Affiliations:** 1 Grupo de Neurocomputación Biológica, Dpto. de Ingeniería Informática, Escuela Politécnica Superior, Universidad Autónoma de Madrid, 28049 Madrid, Spain; 2 The Whitney Laboratory for Marine Bioscience, University of Florida, 9505 Ocean Shore Blvd., St. Augustine, FL 32080, USA

## 

How sensory information is transformed into effective motor action is one of the most fundamental questions in neuroscience. The intrinsic dynamics of sensory networks can play an important role in the sensory-motor transformation. However, it is difficult to experimentally assess the study of all the stages present in the processing of a sensory-motor transformation. Biophysical models of sensory, central and motor systems can largely contribute to understand the information processing mechanisms involved in this transformation. Nevertheless, because of the lack of experimental results, there are very few models including all these stages to address the transformation of sensory dynamics into a motor program.

Complex intrinsic sensory dynamics can be related to multifunctionality in the sensory-motor transformation. Multifunctionality of neural systems has only been partially addressed in neuroscience research. One remarkable example of relationship between intrinsic sensory dynamics and multifunctionality is the gravimetric organ of the mollusk *Clione limacina*[1,2]. In this work we used conductance based models of sensory, central and motor circuits and electrophysiological recordings to address the study of the dual role of a sensory network to organize two different context-dependent motor programs. Our experimental and modeling results indicate that the sensory signals are modified to fit the changing behavioral context, and they are readily interpreted by the rest of the nervous system to produce the correct motor output. We show that a winner-take all dynamics in the gravimetric sensory network drives the repetitive rhythm of *Clione*'s wing CPG model during routine swimming [3]. On the other hand, a winnerless competition dynamics in the same sensory network organizes the irregular pattern observed in the wing CPG during hunting behavior [1]. These two dynamics are interpreted by the wing CPG to generate the characteristic rhythmic motion during routine swimming and the fast irregular motion that is observed during hunting behavior. Our modeling results also indicate that specific activation phase locks in the sensory network dynamics are transformed into specific motor events in the wing CPG. The activation phase locks can play an important role in motor coordination.

These results support the view that the dual dynamics of the statocyst network by itself can explain the two motor programs observed during routine swimming and during hunting behavior in *Clione *[4]. In other words, the motor program could be generated right at the sensory network fitting the changing behavioral context in the sensory signals. In this way, the rest of the neurons in the sensory-motor transformation can just react normally to this signaling.
